# ^18^F-Flotufolastat Positron Emission Tomography in African American Patients With Suspected Prostate Cancer Recurrence: Findings From the Phase 3 SPOTLIGHT Study

**DOI:** 10.1016/j.adro.2024.101571

**Published:** 2024-07-14

**Authors:** Soroush Rais-Bahrami, Mark Fleming, Benjamin Gartrell, William C. Lavely, Albert Chau, Phillip Davis, David M. Schuster

**Affiliations:** a Department of Urology, University of Alabama at Birmingham Heersink School of Medicine, Birmingham, Alabama; b Department of Radiology, University of Alabama at Birmingham Heersink School of Medicine, Birmingham, Alabama; c O'Neal Comprehensive Cancer Center, University of Alabama at Birmingham Heersink School of Medicine, Birmingham, Alabama; d Virginia Oncology Associates, Sarah Cannon Research Institute, Norfolk, Virginia; e Departments of Oncology and Urology, Montefiore Medical Center, New York, New York; f Northside Radiology Associates, Atlanta, Georgia; g Blue Earth Diagnostics Ltd, Oxford, United Kingdom; h Blue Earth Diagnostics Inc., Monroe Township, New Jersey; i Division of Nuclear Medicine and Molecular Imaging, Department of Radiology and Imaging Sciences, Emory University, Atlanta, Georgia

## Abstract

**Purpose:**

Although African American (AA) patients are disproportionately affected by prostate cancer, they are often underrepresented in oncology clinical trials. The SPOTLIGHT study (NCT04186845) assessed the novel diagnostic positron emission tomography radiopharmaceutical, ^18^F-flotufolastat (^18^F-rhPSMA-7.3), in patients with recurrent prostate cancer. The proportion of AA patients enrolled in SPOTLIGHT (17%) was greater than typically enrolled in oncology trials (8.5%) and was representative of the US population (14%). This post hoc analysis of SPOTLIGHT evaluates the diagnostic performance of ^18^F-flotufolastat in AA patients.

**Methods and Materials:**

Patients underwent positron emission tomography/computed tomography 50 to 70 minutes after intravenous administration of 296 MBq ^18^F-flotufolastat. Three blinded readers evaluated all images, with the majority read (agreement of ≥2 readers) result reported here. Standard of truth (SoT) was established with histopathology or correlative imaging. Data from AA patients were evaluated to determine the ^18^F-flotufolastat overall detection rate (DR), positive predictive value (PPV), and verified DR (VDR). VDR (SoT-verified) is equivalent to DR × PPV.

**Results:**

In total, 61 of 366 (17%) patients were AAs. Although baseline characteristics were broadly similar, fewer AA patients (56%) had undergone prostatectomy than non-AA patients (82%). The patient-level DR was 93% (57/61) in AA patients, increasing from 67% at prostate-specific antigen <0.5 ng/mL to 100% at prostate-specific antigen ≥10 ng/mL. Patient-level DR was marginally lower in all other patients (87%, 264/305). However, when stratifying by prior treatment, DRs were similar across ethnic groups in postprostatectomy patients, but in patients with intact prostates, AA patients had higher prostate DR than non-AA patients. SoT-verification (predominantly with conventional imaging [79%]) gave a VDR of 64% and PPV of 68% in AA patients, versus 55% and 64%, respectively, in all other patients.

**Conclusions:**

^18^F-Flotufolastat DRs were marginally higher in AA patients than in all other patients enrolled in SPOTLIGHT. High VDR and PPV were also achieved in AA patients from across all participating centers, indicating the broad applicability of newly US Food and Drug Administration–approved ^18^F-flotufolastat to the US population as a whole.

## Introduction

African Americans comprise the third largest ethnic group in the United States, accounting for approximately 14% of the total population in 2020.^1^ The published literature consistently reports that African American patients have the lowest survival rate of any racial or ethnic group for most cancers.^2^ Prostate cancer is the most commonly diagnosed cancer among African American men in the United States, accounting for 37% of all the newly diagnosed cancers in African Americans in 2022.^2^ Coupled with a genetic predisposition and disparities in disease biology, the suboptimal treatment or restricted access to care that African American patients often experience in comparison with White patients means that they are more than twice as likely to die from prostate cancer compared to White men.^2^, ^3^, ^4^

Despite the increased prevalence of cancer and poorer prognosis among African American patients, data indicate that African American patients continue to be underrepresented in oncology clinical trials, accounting for only 8.5% of enrolled subjects from 2010 to 2021, despite representing 14% of the population.^1^^,^^5^ There have been many calls in recent years, including from the American Society of Clinical Oncology, and the US Food and Drug Administration,^6^^,^^7^ to address this disparity by fostering increased diversity in oncology clinical trial enrollment.

The SPOTLIGHT study (NCT04186845) assessed the diagnostic performance and safety of ^18^F-flotufolastat (^18^F-rhPSMA-7.3), a now US Food and Drug Administration–approved positron emission tomography (PET) diagnostic radiopharmaceutical, in men with biochemical recurrence of prostate cancer following curative-intent treatment.^8^^,^^9^ Data from the phase 3, prospective, multicenter, open-label, single-arm SPOTLIGHT study show high affinity, prostate-specific membrane antigen–targeting ^18^F-flotufolastat to be well tolerated and to offer a clinically meaningful verified detection rate (VDR) for the localization of recurrent prostate cancer.^8^ The proportion of African Americans among the evaluable patients enrolled in SPOTLIGHT was approximately 17%, almost twice the proportion typically reported in oncology clinical trials,^5^ and closer to a representative proportion of the US population.^1^

Herein, we present a post hoc subgroup analysis of the SPOTLIGHT study to establish the diagnostic performance of ^18^F-flotufolastat in African American patients with biochemical recurrence of prostate cancer to evaluate the hypothesis that ^18^F-flotufolastat is broadly applicable to the US population as a whole.

## Methods and Materials

Patients with elevated prostate-specific antigen (PSA) suspicious for recurrence of previously treated, localized prostate cancer who were being considered for potential curative-intent salvage therapy were enrolled as previously described.^8^ An elevated PSA clinically suspicious for recurrence was defined as ≥0.2 ng/mL with a subsequent confirmatory value ≥0.2 ng/mL for patients after radical prostatectomy (±adjuvant therapy), or as nadir +2 ng/mL following radiation therapy, brachytherapy, or focal gland therapy.^8^ The patients underwent PET/computed tomography 50 to 70 minutes after delivery of 8 mCi, 296 MBq ± 20% ^18^F-flotufolastat as an intravenous bolus. For standard of truth (SoT) verification, patients either had conventional imaging (functional or multiparametric magnetic resonance imaging, computed tomography, ^18^F-fluciclovine PET, or ^18^F-NaF PET as per site standard of care) within 90 days of the ^18^F-flotufolastat PET, or where safe and medically feasible, the most accessible PET-positive lesion in 1 of 3 defined regions (prostate/prostate bed, pelvic lymph nodes, other extrapelvic sites [bone, extrapelvic nodes, viscera, and other soft tissues]) was biopsied under radiological guidance within 60 days of the ^18^F-flotufolastat PET. All ^18^F-flotufolastat images were interpreted by 3 blinded independent central readers. The majority read result represents agreement of 2 or more readers.

### Statistical analysis

The efficacy analysis population (EAP) comprised all patients who underwent ^18^F-flotufolastat PET and had data for SoT assessment.^8^ For the present analysis, data from all patients across all European and the US sites who were included in the EAP were evaluated in order to report the overall detection rate, the VDR, and the positive predictive value (PPV) of ^18^F-flotufolastatat by majority read at both a patient-level and region-level among Black or African American patients. The impact of prior treatment and clinical factors such as PSA level at baseline and International Society for Urological Pathology (ISUP) grade group on the diagnostic performance were explored. For this analysis, the overall detection rate is defined as the number of patients with ≥1 PET-positive lesion divided by the number of patients in the cohort (percent positivity). VDR is defined as the percentage of all patients with ≥1 true-positive (according to available SoT) PET detection, regardless of any coexisting false positives. The denominator is all patients scanned (PET-positive and PET-negative). Finally, PPV represents the number of true-positive patients divided by the number of PET-positive patients (both true positive and false positive), thus does not include PET-negative patients.

## Results

**Figure 1:**
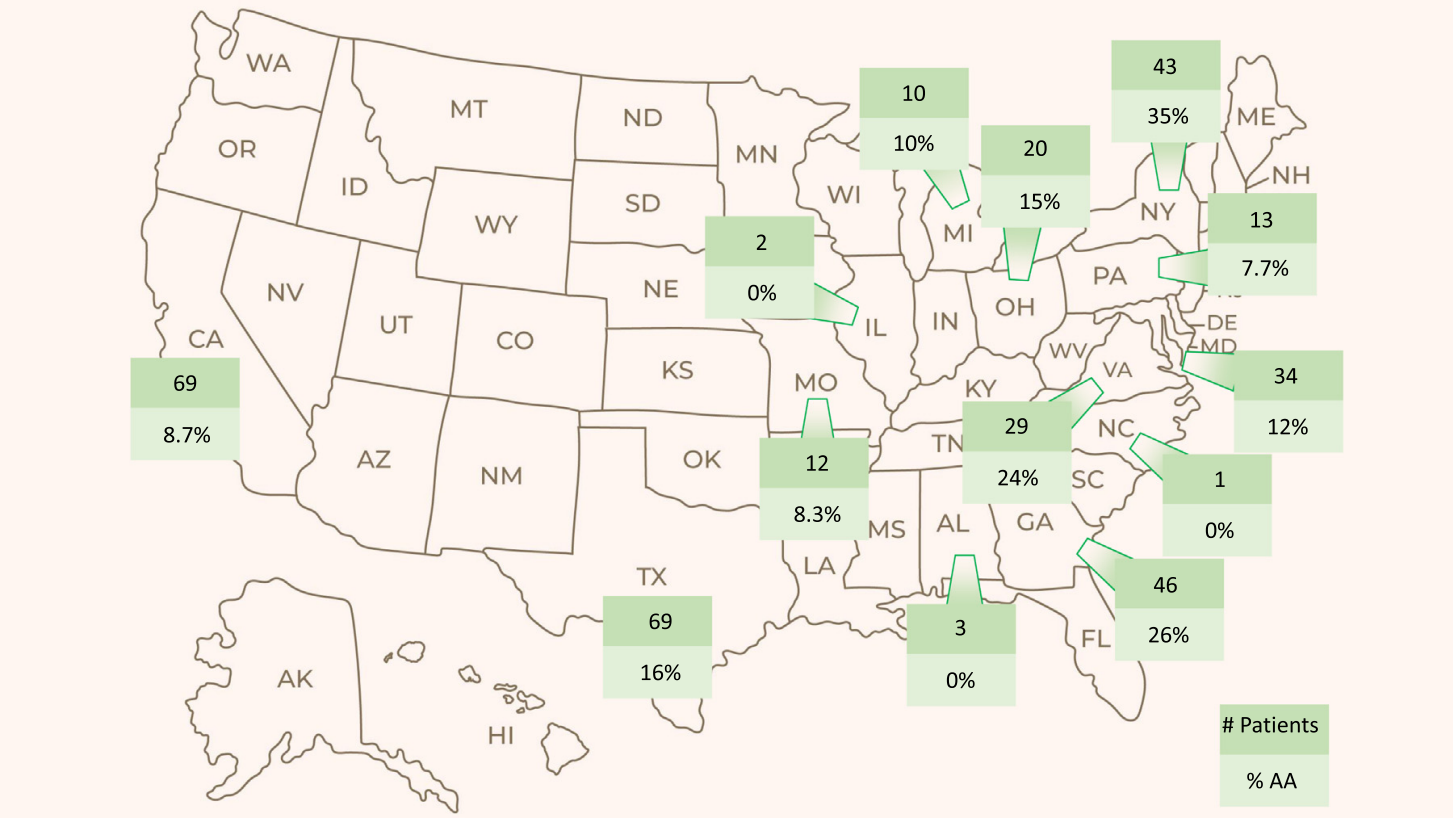
Total number and proportion of African American (AA) patients enrolled in the SPOTLIGHT efficacy analysis population (EAP) presented per US state. Green boxes indicate states from which SPOTLIGHT EAP patients were enrolled in the United States. The upper number denotes the total number of patients enrolled from that state, and the lower figure reports the proportion of these who were AA. An additional 15 patients were recruited from Europe, none of whom were Black. *Abbreviations:* AK = Alaska; AL = Alabama; AZ = Arizona; CA = California; CO = Colorado; DE = Delaware; FL = Florida; GA = Georgia; HI = Hawaii; ID = Idaho; IL = Illinois; IN = Indiana; KS = Kansas; KY = Kentucky; LA = Louisiana; MD = Maryland; ME = Maine; MI = Michigan; MN = Minnesota; MO = Missouri; MS = Mississippi; MT = Montana; NC = North Carolina; ND = North Dakota; NE = Nebraska; NH = New Hampshire; NM = New Mexico; NV = Nevada; NY = New York; OH = Ohio; OK = Oklahoma; OR = Oregon; PA = Pennsylvania; SC = South Carolina; SD = South Dakota; TN = Tennessee; TX = Texas; UT = Utah; VA = Virginia; WA = Washington; WI = Wisconsin; WV = West Virginia; WY = Wyoming.

In total, 61 (17%) of the 366 patients in the EAP were categorized as Black or African American. All 61 were recruited from sites in the United States. Figure 1 presents the proportion of African American patients enrolled per US state. Baseline characteristics of African American patients were similar to those of the overall population (Table 1); however, fewer African American patients (56%) had undergone radical prostatectomy compared with all other (non-African American) patients enrolled in SPOTLIGHT (82%), and the overall study population (77%).

**Table 1:** Patient characteristics

	Patients in the EAP (N = 366)		
Patient characteristic	All patients (N =366)*	African American patients (n = 61)	All other (non-African American) patients (n = 305)
Age (y)			
Mean	68.4	68.3	68.5
SD	7.86	8.06	7.83
Range	43-85	53-85	43-85
Gleason score, n (%)			
≤6	39 (11)	11 (18)	28 (9.2)
7	218 (60)	35 (57)	183 (60)
≥8	95 (26)	12 (20)	83 (27)
Missing	14 (3.8)	3 (4.9)	11 (3.6)
ISUP grade group, n (%)			
1	39 (11)	11 (18)	28 (9.2)
2	95 (26)	16 (26)	79 (26)
3	111 (30)	18 (30)	93 (30)
4	36 (10)	9 (15)	27 (8.9)
5	59 (16)	3 (4.9)	56 (18)
Missing	26 (7.1)	4 (6.6)	22 (7.2)
Time from initial prostate cancer diagnosis (mo)			
Median	70	110	69
Range	2-409	8-319	2-409
Prior therapy, n (%)			
With prior prostatectomy	283 (77)	34 (56)	249 (82)
With radiation therapy	126 (45)	18 (53)	108 (43)
Without radiation therapy	157 (55)	16 (47)	141 (57)
Without prior prostatectomy	83 (23)	27 (44)	56 (18)
Radiation therapy	75 (90)	25 (93)	50 (89)
Other therapy	7 (8.4)	1 (3.7)	6 (11)
No other therapy	1 (1.2)	1 (3.7)	0 (0.0)
Baseline PSA (ng/mL)†			
All patients in subgroup	N = 366	n = 61	n = 305
Median	1.27	3.20	0.94
Range	0.03-134.6	0.20-32.9	0.03-134.6
All patients with prior prostatectomy	n = 283	n = 34	n = 249
Median	0.70	2.00	0.67
Range	0.10-32.2	0.20-24.7	0.10-32.2
All patients without prior prostatectomy	n = 83	n = 27	n = 56
Median	4.70	5.22	4.41
Range	0.03-134.6.0	2.2-32.9	0.03-134.6

*Abbreviations:* EAP = efficacy analysis population; ISUP =International Society for Urological Pathology; PSA = prostate-specific antigen.

⁎ Data as reported by Jani et al.^8^

† Two patients in the SPOTLIGHT efficacy analysis population were found on re-evaluation to have a prescan prostate-specific antigen value that did not meet the inclusion criterion. These patients were subsequently excluded from the per protocol population as previously reported.^8^

### Overall ^18^F-flotufolastat detection rate in African American patients

The majority read patient-level detection rate was high among African American patients, with 93% of patients (57/61; 95% CI, 84.1%-98.2%) found to have a positive ^18^F-flotufolastat scan. This was marginally higher than the rate among all other patients (87% [264/305]; 95% CI, 82.2%-90.2%). By region, 59% (36/61; 95% CI, 45.7%-71.4%) of African American patients were found to have ^18^F-flotufolastat-avid lesions in the prostate/prostate bed, 25% (15/61; 95% CI, 14.5%-37.3%) in pelvic lymph nodes, and 38% (23/61; 95% CI, 25.6%-51.0%) in extrapelvic anatomic sites.

#### Impact of PSA and ISUP grade group

**Figure 2:**
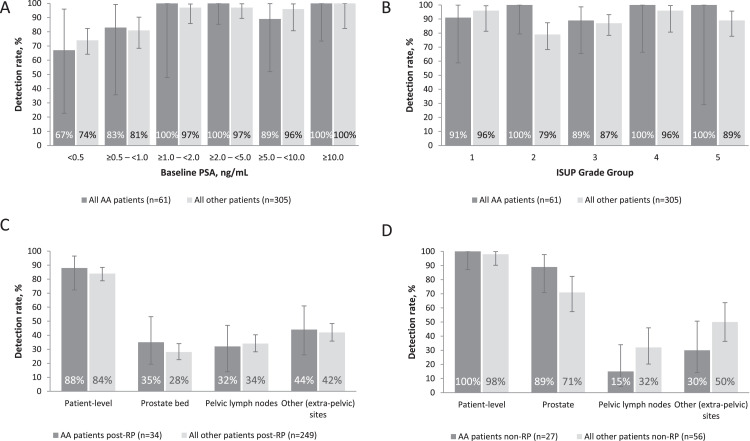
^18^F-Flotufolastat overall patient-level detection rates in African American (AA; n = 61) and all other (non-AA) patients (n = 305) stratified by (A) baseline PSA, (B) International Society for Urological Pathology (ISUP) grade group*, and (C and D) prior treatment. Panel C presents data from AA patients (n = 34) and all other (non-AA) patients (n = 249) who had previously undergone radical prostatectomy (RP), and panel D from AA patients (n = 27) and all other (non-AA) patients (n = 56) who had not previously undergone RP. *Abbreviation:* PSA = prostate-specific antigen. *ISUP grade group data were not available for 4 AA patients and 22 other patients.

As previously reported for the overall SPOTLIGHT population,^8^ detection rates among African American patients broadly increased with increasing PSA from 67% (4/6; 95% CI, 22.3%-95.7%) at PSA <0.5 ng/mL to 100% (12/12; 95% CI, 73.5%-100.0%) at PSA ≥10 ng/mL. Similar rates were observed among all other (non-African American) patients as shown in Fig. 2A.

Although the small number of patients in some groups limited the analyses, the overall ^18^F-flotufolastat detection rate was high across all ISUP grade groups, irrespective of ethnicity (Fig. 2B).

#### Impact of prior treatment

Given the lower rates of radical prostatectomy among African American patients, we further evaluated detection rates stratified by prior treatment (Fig. 2). Among the 283 patients who had previously undergone radical prostatectomy, the overall patient-level ^18^F-flotufolastat detection rate was 88% (30/34; 95% CI, 72.5%-96.7%) in African American patients and 84% (209/249; 95% CI, 78.8%-88.3%) among all other (non-African American) patients. As shown in Fig. 2C, the regional detection rates after prostatectomy were similar in African American patients to those in all other patients. Among the 83 patients who had not undergone radical prostatectomy the overall patient-level ^18^F-flotufolastat detection rate was 100% (27/27; 95% CI, 87.2%-100.0%) in African American patients and 98% (55/56; 95% CI, 90.4%-100.0%) among all other patients. There were some small differences in regional detection rates between African American patients and all other patients with intact prostates. As shown in Fig. 2D, detection rates among African Americans were marginally lower in the prostate, but higher in all extraprostatic regions.

### ^18^F-Flotufolastat VDR

In total, 13 (21%) of the 61 African American patients had histopathology available for SoT assessment, with the remaining 48 (79%) having only conventional imaging available for categorization of PET-positive lesions as true positive or false positive. A similar proportion (56/305; 18%) of all other (non-African American) patients had histopathology available for SoT.

Following SoT assessment, true-positive lesions were observed in 39 African American patients, leading to a patient-level VDR of 64% (95% CI, 50.6%-75.8%), higher than the VDR of 55% (95% CI, 49.6%-61.1%) observed among all other patients. A notable increase in the patient-level VDR was observed when focusing on only the subgroup of African American patients with the gold standard histopathology available as a SoT (n = 13). Among these patients, the patient-level VDR increased to 77% (95% CI, 46.2%-95.0%).

**Figure 3:**
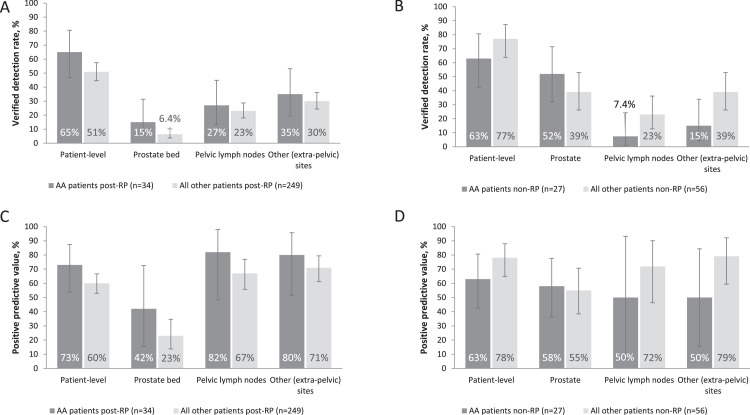
^18^F-Flotufolastat overall patient-level and regional verified detection rates and positive predictive value in African American (AA) and all other (non-AA) patients stratified by prior treatment. (A) Verified detection rates among AA patients (n = 34) and all other (non-AA) patients (n = 249) who had previously undergone radical prostatectomy (RP). (B) Verified detection rates among the AA patients (n = 27) and all other (non-AA) patients (n = 56) who had not previously undergone RP. (C) Positive predictive value in AA patients (n = 34) and all other (non-AA) patients (n = 249) who had previously undergone RP, and (D) Positive predictive value in AA patients (n = 27) and all other (non-AA) patients (n = 56) who had not previously undergone RP.

When stratifying patients by prior treatment, marginally higher patient- and region-level VDR were noted among African American patients who had undergone radical prostatectomy compared with all other patients who had undergone radical prostatectomy (Fig. 3A). Among the patients with intact prostates, African American patients were seen to have higher VDR in the prostate and lower VDR in extraprostatic regions compared with all other patients (Fig. 3B).

Table 2 presents the patient-level VDR stratified by prior treatment and baseline PSA level. VDR broadly increased with increasing PSA levels; however, the low number of patients in some categories limits the comparisons that can be drawn between ethnic groups.

**Table 2:** Patient-level verified detection rate stratified by prior treatment and baseline prostate-specific antigen level

	African American patients			All other patients		
Baseline PSA (ng/mL)	All (n = 61)	Patients with prior prostatectomy (n = 34)	Patients without prior prostatectomy (n = 27)	All (n = 305)	Patients with prior prostatectomy (n = 249)	Patients without prior prostatectomy (n = 56)
<0.5	0%	0%	–	33%	34%	0%
	(0/6)	(0/6)		(33/99)	(33/98)	(0/1)
	95% CI, 0.0%-45.9%	95% CI, 0.0%-45.9%		95% CI, 24.2%-43.5%	95% CI, 24.4%-43.9%	95% CI, 0.0%-97.5%
≥0.5 to <1.0	33%	33%	–	51%	51%	–
	(2/6)	(2/6)		(29/57)	(29/57)	
	95% CI, 4.3%-77.7%	95% CI, 4.3%-77.7%		95% CI, 37.3%-64.4%	95% CI, 37.3%-64.4%	
≥1.0 to <2.0	80%	80%	–	63%	62%	100%
	(4/5)	(4/5)		(24/38)	(23/37)	(1/1)
	95% CI,28.4%-99.5%	95% CI, 28.4%-99.5%		95% CI, 46.0%-78.2%	95% CI, 44.8%-77.5%	95% CI, 2.5%-100.0%
≥2.0 to <5.0	87%	100%	77%	74%	77%	70%
	(20/23)	(10/10)	(10/13)	(48/65)	(27/35)	(21/30)
	95% CI, 66.4%-97.2%	95% CI, 69.2%-100.0%	95% CI, 46.2%-95.0%	95% CI, 61.5%-84.0%	95% CI, 59.9%-89.6%	95% CI, 50.6%-85.3%
≥5.0 to <10.0	44%	67%	33%	67%	54%	79%
	(4/9)	(2/3)	(2/6)	(18/27)	(7/13)	(11/14)
	95% CI, 13.7%-78.8%	95% CI, 9.4%-99.2%	95% CI, 4.3%-77.7%	95% CI. 46.0%-83.5%	95% CI, 25.1%-80.8%	95% CI, 49.2%-95.3%
≥10.0	75%	100%	63%	90%	78%	100%
	(9/12)	(4/4)	(5/8)	(17/19)	(7/9)	(10/10)
	95% CI, 42.8%-94.5%	95% CI, 39.8%-100.0%	95% CI, 24.5%-91.5%	95% CI, 66.9%-98.7%	95% CI, 40.0%-97.2%	95% CI, 69.2%-100.0%

*Abbreviation:* PSA = prostate-specific antigen.

### ^18^F-Flotufolastat patient- and region-level PPV

The overall patient-level PPV in African American patients was 68% (39/57; 95% CI, 54.8%-80.1%), similar to the value obtained among all other patients (64% [169/264]; 95% CI, 57.9%-69.8%). Region-level PPV among African American patients were 53% (19/36; 95% CI, 35.5%-69.6%) in prostate/prostate bed, 73% (11/15; 95% CI, 44.9%-92.2%) in pelvic lymph nodes and 70% (16/23; 95% CI, 47.1%-86.8%) in extrapelvic regions. As with VDR, a higher patient-level PPV (77%; 95% CI, 46.2%-95.0%) was noted among the subgroup of 13 African American patients with the gold standard histopathology available as a SoT.

When stratifying patients by prior treatment, higher patient- and region-level PPV were noted among African American patients who had undergone radical prostatectomy compared with all other patients who had undergone radical prostatectomy (Fig. 3C). Although the small number of patients in some categories limited the data, among the patients with intact prostates, African American patients were seen to have lower PPV in extraprostatic regions compared with all other patients (Fig. 3D).

Table 3 presents the patient-level PPV stratified by prior treatment and baseline PSA level. As with VDR, the patient-level PPV broadly increased with increasing PSA levels; however, comparisons between ethnic groups are limited by the small number of patients in some categories.

**Table 3:** Patient-level positive predictive value stratified by prior treatment and baseline prostate-specific antigen level

	African American patients			All other patients		
Baseline PSA (ng/mL)	All (n = 61)	Patients with prior prostatectomy (n = 34)	Patients without prior prostatectomy (n = 27)	All (n = 305)	Patients with prior prostatectomy (n = 249)	Patients without prior prostatectomy (n = 56)
<0.5	0%	0%	–	45%	45%	–
	(0/4)	(0/4)		(33/73)	(33/73)	
	95% CI, 0.0%-60.2%	95% CI, 0.0%-60.2%		95% CI, 33.5%-57.3%	95% CI, 33.5%-57.3%	
≥0.5 to <1.0	40%	40%	–	63%	63%	–
	(2/5)	(2/5)		(29/46)	(29/46)	
	95% CI, 5.3%-85.3%	95% CI, 5.3%-85.3%		95% CI, 47.5%-76.8%	95% CI, 47.5%-76.8%	
≥1.0 to <2.0	80%	80%	–	65%	64%	100%
	(4/5)	(4/5)		(24/37)	(23/36)	(1/1)
	95% CI, 28.4%-99.5%	95% CI, 28.4%-99.5%		95% CI, 47.5%-79.8%	95% CI, 46.2%-79.2%	95% CI, 2.5%-100.0%
≥2.0 to <5.0	87%	100%	77%	76%	82%	70%
	(20/23)	(10/10)	(10/13)	(48/63)	(27/33)	(21/30)
	95% CI, 66.4%-97.2%	95% CI, 69.2%-100.0%	95% CI, 46.2%-95.0%	95% CI, 63.8%-86.0%	95% CI, 64.5%-93.0%	95% CI, 50.6%-85.3%
≥5.0 to <10.0	50%	100%	33%	69%	58%	79%
	(4/8)	(2/2)	(2/6)	(18/26)	(7/12)	(11/14)
	95% CI, 15.7%-84.3%	95% CI, 15.8%-100.0%	95% CI, 4.3%-77.7%	95% CI, 48.2%-85.7%	95% CI, 27.7%-84.8%	95% CI, 49.2%-95.3%
≥10.0	75%	100%	63%	90%	78%	100%
	(9/12)	(4/4)	(5/8)	(17/19)	(7/9)	(10/10)
	95% CI, 42.8%-94.5%	95% CI, 39.8%-100.0%	95% CI, 24.5%-91.5%	95% CI, 66.9%-98.7%	95% CI, 40.0%-97.2%	95% CI, 69.2%-100.0%

*Abbreviation:* PSA = prostate-specific antigen.

## Discussion

The SPOTLIGHT study evaluated the diagnostic performance and safety of prostate-specific membrane antigen–targeting radiopharmaceutical, ^18^F-flotufolastat, in a large multi-institutional population of men from across the United States and Europe who had recurrent prostate cancer.^8^ Given the high prevalence and high mortality of prostate cancer in African American men,^2^^,^^3^ the present post hoc analysis was conducted to evaluate the performance of ^18^F-flotufolastat in African American patients enrolled in SPOTLIGHT.

African American patients accounted for just 8.5% of participants in US clinical trials between 2010 and 2021, despite representing 14% of the population.^1^^,^^5^ Many reasons have been proposed to account for this disparity. Financial barriers such as medical and/or indirect costs like travel or missed work are more likely to prevent participation among ethnic minority populations, because they often have lower socioeconomic status relative to the White population.^7^ Additionally, the limited awareness of, and access to, clinical trials is also linked to a lower number of African American participants,^10^ particularly in cases where study exclusion criteria inadvertently exclude African American patients by not accounting for physiological differences compared with White patients, such as testosterone levels or renal functional thresholds.^10^^,^^11^

As well as further contributing to racial disparities in cancer outcomes, oncology clinical trials with low diversity populations ultimately yield results that are less useful to population-wide clinical decision-making than higher inclusivity trials. In order to understand potential differences in efficacy and safety across diverse populations, and to mitigate ethnic disparities in cancer care and outcomes, encouraging inclusive participation in oncology clinical trials is crucial. Although there was no predetermined strategy around enrollment of specific ethnic groups, the SPOTLIGHT study enrolled a proportion of African American patients that was representative of the US population. The data presented in Fig. 1 indicate that the greatest proportion of African American patients was recruited from eastern seaboard states, particularly New York, Georgia, and Virginia. Future prostate cancer clinical studies aiming to enroll higher numbers of African American patients might look to target recruitment from these sites with larger African American populations, or will need to institute international recruitment of underrepresented minorities in other regions.

Given the diversity of its patient population, the main findings from SPOTLIGHT^8^ should be applicable to the US population as a whole. Nevertheless, the current post hoc analysis provides further encouraging evidence for the promising diagnostic performance of ^18^F-flotufolastat among African American patients with biochemical recurrence of prostate cancer. Over 90% of African American patients enrolled in SPOTLIGHT had 1 or more ^18^F-flotufolastat-avid lesion. This is marginally higher than among all other (non-African American) patients (87%, 264/305). However, some differences were noted in the clinical characteristics of African American patients compared with all other enrolled patients that likely contribute to this marginally increased detection rate among African American patients. Consistent with previously published data,^12^^,^^13^ fewer African American patients enrolled in SPOTLIGHT had undergone radical prostatectomy compared with the remainder of enrolled patients (56% vs 82%), and while this may suggest a treatment disparity, it may be a reflection of African American patients presenting with more advanced disease, and, indeed, Ellis et al's^13^ data suggest there to be no race disparity in guideline-concordant care. Likely a consequence of the PSA enrollment thresholds being based on prior treatment,^8^ and reflecting their lower rate of radical prostatectomy, we observe a higher median PSA level in African American patients when compared with the other patients in SPOTLIGHT. As shown here and previously,^8^ the detection rate of ^18^F-flotufolastat increases with increasing PSA level, which may account for the marginally increased detection rate in African American patients when compared with all other patients in SPOTLIGHT. As seen in Fig. 2, when detection rates are stratified by baseline PSA, ethnicity has little impact on ^18^F-flotufolastat detection rates.

To further explore the impact of prior treatment, all patients were stratified into groups according to whether or not they had undergone radical prostatectomy. Interestingly, the data show that in patients with an intact prostate, African American patients had marginally higher detection rates in the prostate and lower extraprostatic detection than non-African American patients.

When using a SoT method to verify ^18^F-flotufolastat-positive lesions, we observe that the patient-level VDR is also higher among African American patients (64%) than among all other patients (55%) and the SPOTLIGHT population as a whole (57%).^8^ However, as reported by Jani et al,^8^ data were limited by the relatively high number of patients who had only conventional imaging available for SoT. It is likely that a number of lesions categorized as false positive in the trial would have been categorized true positive if a more accurate and sensitive SoT method had been used.^8^ When restricting to only patients with the gold standard histopathology (n = 13, 21% of African American patients), the VDR in African American patients (77%) is similar compared to that reported for the overall population (81%^8^), as is the patient-level PPV metric (77% vs 82%^8^).

Although we observe a marginally increased detection rate among African American patients compared with all other patients based on the point estimates in the respective groups, there were no prespecified statistical analyses in this post hoc secondary analysis and so future prospective studies would be needed to confirm any differences between ethnic groups.

In conclusion, while geographic targeting of regions with populations high in African Americans may serve to address the known disparities of enrollment of African American patients in future oncology clinical trials, we show here that adequate representation is achievable. ^18^F-Flotufolastat showed a marginally higher detection rate for recurrent prostate cancer in African Americans compared with all other patients. Moreover, verification of these imaging findings results in a high VDR and PPV in this population that are in line with data observed in the overall population of SPOTLIGHT patients from across the United States and Europe. The data evaluated in this study help to demonstrate the broad applicability of newly approved ^18^F-flotufolastat to the US population as a whole.

## Disclosures

Soroush Rais-Bahrami has research funding from the National Institutes of Health/National Cancer Institute, Department of Defense, Blue Earth Diagnostics, Genomic Health Inc, Astellas, and Progenics. He serves as a consultant to Philips Corp, Blue Earth Diagnostics, Genomic Health Inc, Bayer Healthcare, Intuitive Surgical, Sanofi/Genzyme, Progenics, GE Healthcare, and Boston Scientific. Mark Fleming has received travel support from Blue Earth Diagnostics. Benjamin Gartrell has received consulting fees from Janssen, Genzyme, Aveo, Pfizer, Seagen, and Blue Earth Diagnostics. William C. Lavely has received speaker's fees from Lantheus and GE Healthcare. Albert Chau received consultancy fees from Blue Earth Diagnostics Ltd for data management and statistical services. Phillip Davis is an employee of Blue Earth Diagnostics Inc. David M. Schuster has acted as a consultant for Global Medical Solutions Taiwan, Progenics Pharmaceuticals Inc, Heidelberg University, and DuChemBio Co Ltd. He participates through the Emory Office of Sponsored Projects in full compliance with Emory University sponsored research and conflict of interest regulations in sponsored grants including those funded or partially funded by Blue Earth Diagnostics Ltd, Nihon Medi-Physics Co Ltd, Telix Pharmaceuticals (United States) Inc, Advanced Accelerator Applications, FUJIFILM Pharmaceuticals U.S.A. Inc, and Amgen Inc. He participates in educational initiatives with School of Breast Oncology and PrecisCa and provides medicolegal consulting vetted through Emory School of Medicine.
